# Theoretical analysis of the effect of isotropy on the effective diffusion coefficient in the porous and agglomerated phase of the electrodes of a PEMFC

**DOI:** 10.1038/s41598-024-57846-w

**Published:** 2024-03-27

**Authors:** C. Pacheco, Romeli Barbosa, A. Navarro-Montejo, L. C. Ordoñez

**Affiliations:** Centro de Investigación Científica de Yucatán, Parque Científico y Tecnológico de Yucatán, Carretera Sierra Papacal–Chuburna Puerto, km 5, Sierra Papacal, 97302 Mérida, Yucatán Mexico

**Keywords:** Effective transport coefficient, PEMFC, Fick's diffusion, Catalyst layer, Agglomerates, Fuel cells, Fuel cells, Fluidics, Two-dimensional materials

## Abstract

In polymer membrane fuel cells (PEMFC), the pore microstructure and the effective diffusion coefficient ($D_{eff}$) of the catalytic layer have a significant impact on the overall performance of the fuel cell. In this work, numerical methods to simulate PEMFC catalytic layers were used to study the effect of isotropy ($I_{xy}$) on the $D_{eff}$. The proposed methodology studies reconstructed systems by Simulated Annealing imaging with different surface fractions of microstructures composed by two diffusive phases: agglomerates and pores. The $D_{eff}$ is determined numerically by the Finite Volume Method solved for Fick's First Law of Diffusion. The results show that the proposed methodology can effectively quantify the effect of isotropy on the $D_{eff}$ for both diffusion phases. Two trends were obtained in the magnitude of the $D_{eff}$ concerning the change in isotropy: (1) an analytical equation is proposed in this article for $D_{eff} \ge 5\% D_{0}$ and (2) numerical solutions are determined for $D_{eff} < 5\% D_{0} .$ In our analytical equation are both a lineal and a logarithmic sweep. When the surface fraction is $\emptyset =$ 50%, the $D_{eff}$ decreases more linearly than $\emptyset = 10\%$ at the beginning of the isotropy change, which indicates that small changes in isotropy in the particulate material modify it drastically; under these conditions the diffusion coefficient in the pore is predominant. (3) When the surface fraction is less than 50%, the $D_{eff}$ decreases more exponentially at the beginning and more linearly at the end of the isotropy change, which shows that small isotropy changes in the bar-aligned material drastically alter it. In this trend, diffusion in the agglomerate is less affected by isotropy. The proposed methodology can be used as a design tool to improve the mass transport in porous PEMFC electrodes.

## Introduction

The urgent need to reduce pollution and carbon dioxide in the atmosphere has increased the interest in cleaner and more efficient systems^1–3^. Proton Exchange Membrane Fuel Cells (PEMFC) are expected to play a critical role in sustainable energy production in the near future as they promise to be energy conversion devices for applications such as automobiles, trains, portable electronic devices, and others due to their high efficiency, fast start-up, and zero emissions when fueled with hydrogen^4–7^.

However, this technology still faces challenges; one of them is related to mass transport in the agglomerates and porous media, as the performance of a PEMFC is mainly constrained by mass transport limitations. In addition to the effective transport of electrons, ions, and reactant gases are prerequisites for electrochemical reactions to occur^8–12^. To improve this mechanism, a detailed understanding of the relationship between the properties of the agglomerates and the porous media is required.

To comprehend mass transport in agglomerates and the porous media, one must be aware of three crucial phenomena: molecular diffusion, convective diffusion, and Knudsen diffusion^13,14^. Molecular diffusion refers to the transport of chemical species under concentration gradients (Fick Diffusion)^15^. Convection occurs under pressure gradients^16^. Knudsen diffusion applies to the displacement of flow or gases on small length scales^17,18^. These three phenomena can be examined at the agglomerates, pore level, or macro scales. However, pore-scale models must be used to account for the structural details of porous media. Furthermore, transport phenomena are linked to the heterogeneous morphology of the components that comprise the catalytic layer (CL), ionomer, and pores^19,20^.

The catalytic layer is a crucial component of the PEMFC, where the electrochemical reaction rates are controlled. La CL consists of a void region, ionomer, and platinum carbon-supported catalyst (Pt/C)^21^. The transport phenomena and characteristics of the CL depend on the composite morphological structures, compositions and pore structure (closed pores and dead-end pores)^22,23^.

Significant progress has been made in recent years in the CL simulation of PEMFC. However, it remains a challenge to evaluate transport in porous media. Nonetheless, various studies have suggested evaluations of pore diffusion behavior. In a recent study, Shin et al.^24^ proposed an effective utilization factor of the CL related to the tortuosity of the pathways for reagent mass transport within the CL. To simulate the mass transport of reactants within the CLs, this study utilized the Boltzmann method. Numerous studies have reported that Boltzmann method is useful for simulating mass transport phenomena inside heterogeneous porous media^25–27^. Lange et al.^28^ showed that the effect of Fick diffusion on CL proved to be significant in numerical estimation. They used stochastic reconstruction to obtain the CL pore distribution and evaluated the effective Diffusion-coefficient for a range of porosity ($\emptyset$) from 25 to 50%; they found that the oxygen diffusion coefficient is in the range of 0.0025–0.02 cm^2^ s^−1^. They concluded that the diffusion transport limitations are underestimated if the Fick diffusion is not considered. The same effect is reported by Yu & Carter^29^ their calculations of the diffusion coefficient of the whole electrode (gas diffusing layer and CL) showed that ignoring the Fick diffusion underestimates the diffusion limitations. J.O. Ceballos et al.^30^ present several numerical simulations of the PEM fuel cell and analyze the effect of tortuosity on its overall performance. They present polarization curves evaluated with different effective diffusion models in the anode and cathode. They reported different current density values when employing the various diffusion models. Congfan Zhao et al.^31^ studied the effect of different catalyst layer designs used in PEMFC using stochastic reconstruction methods. The generation of the catalytic layer consists of 4 phases, firstly, a skeleton of carbon particles, then the generation of Pt particles on the carbon surface, followed by the addition of ionomer. The last phase consists of growing the microstructure with the complete elements. The results presented demonstrate that the fabrication of the MEA using the conventional process with the consideration of carbon selection, porosity, dispersion and ionomer content is of great benefit for the efficient use of Pt in the CL.

On the other hand, several studies have found that the CL can be analyzed as a random heterogeneous material (RHM)^32–35^; RHMs can be characterized by an Effective Transport Coefficient (ETC), which measures the material’s ability to transport physical properties, and which is affected by the properties of the phases composing the CL layer. Stochastic reconstruction allows defining the microstructure in a mesh of nodes to study the ETC in more detail^34,36^.

Strategies for the study of RHM have been previously published by our research group. Rodriguez et al.^35^ analyzed the effect of size reduction on microstructure ETC´s and implemented reconstruction using the SA method. Barbosa et al.^36^ developed a scaling method to determine the ETC´s in RHM using stochastic reconstruction techniques and analytical techniques. Pacheco et al.^37^ reported on the effect of topological entropy on the ETC´s in composites aligned and reconstructed by SA. In this research work, the use of stochastic reconstruction using the SA method is employed to analyze the behavior of oxygen diffusion through the ionomer inside the agglomerate and the void space that forms in the CL microstructures that start with an aligned bar architecture and are reconstructed to obtain a dispersed random material. The novelty of our proposal consists of understanding the influence of isotropy on the oxygen diffusion coefficient in both porous and the agglomerate medium to design or analyze catalyst layers of PEMFC. The use of the numerical o theoretical results allows making decisions about the modification of the geometric structure of the materials. Considering in decision making a change of the isotropy or a change of surface fraction. This also helps to improve the accuracy of models and numerical simulations, allowing the optimization of the overall processes. The limitations of simulated annealing found in this study are the boundary conditions, specifically the edge effects since the pixels at the edges result in unwanted effects due to the lack of neighboring pixels in certain directions. This limitation was solved by implementing strategies in the code that allow preserving the continuity of the microstructures without unwanted border effects. The samples studied comprise domains of 350 × 350 pixels where each pixel is equivalent to 0.1 µm, so the samples studies in this work are 35 µm × 35 µm. The evolution of the isotropy and the $D_{eff}$ in the SA agitation steps are systematically evaluated to allow the design of better CLs. The results are obtained using the working group's computational algorithms, which are programmed in C language and developed using the Dev-C +  + compiler (Company Free Software Foundation, Inc. Version 5.11).

## Methods

**Figure 1:**
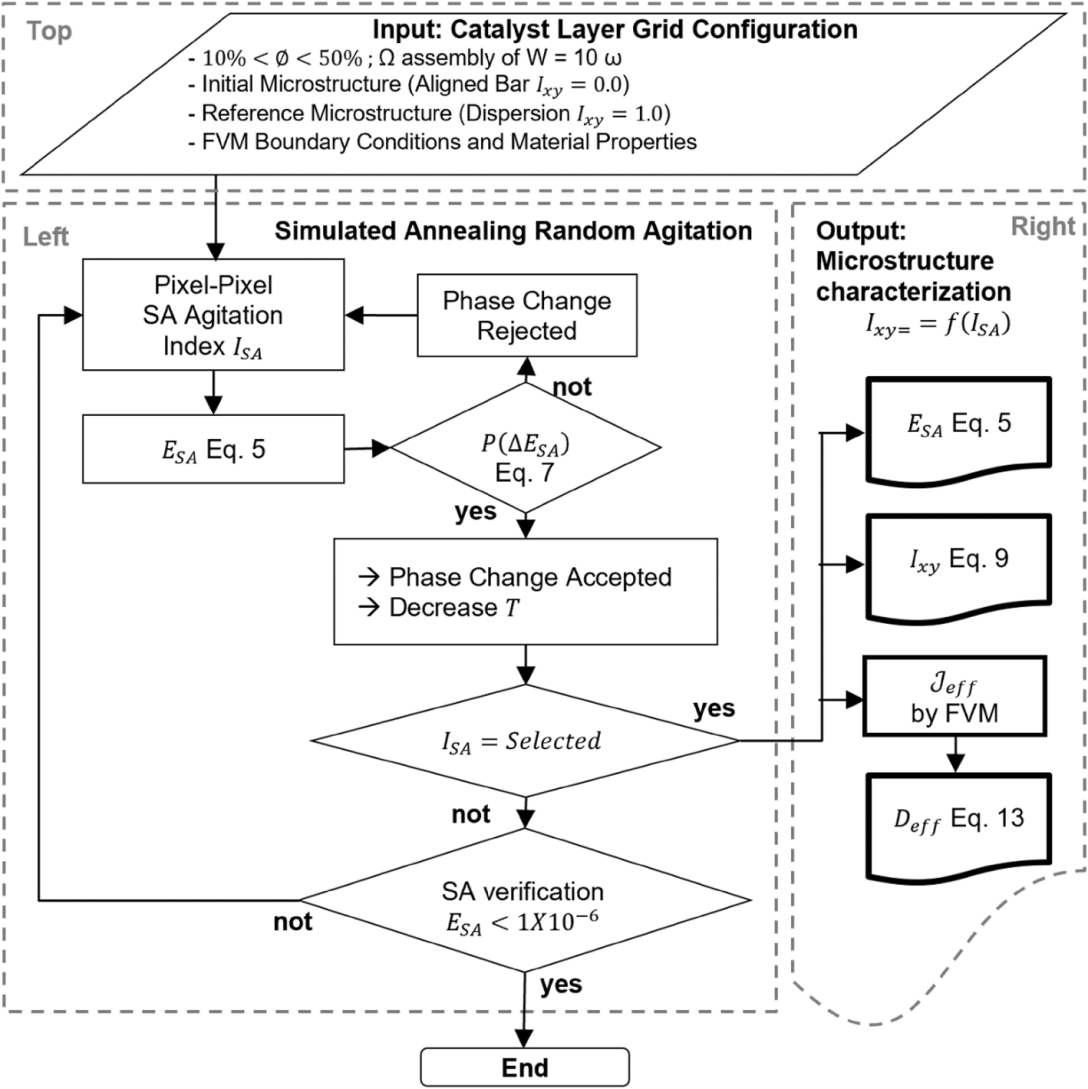
Block diagram of the implemented methodology.

The scope of this work is constrained to isotropic biphasic agglomerates, specifically those consisting of a randomly distributed phase (Pt/C catalyst) within a matrix phase (ionomer). The digital system is spatially defined by a mesh of control volumes (CV). These CV have recognizable phases and diffusivity coefficient values defined at each node. The SA method^35,37,38^ is used to stir the initial system into a reference system sequentially. The materials are statistically characterized by intrinsic correlation functions in the SA reconstruction methodology. Because of this, it is possible to determine the isotropy ($$I_{xy} )$$ at a specific SA stirring interval, defined by the iteration number of the SA method ($$I_{SA}$$). The $$D_{eff}$$ is established by the FVM and is relates to $$I_{xy}$$ of the current system. A block diagram of the methodology proposed in this research is presented in Fig. 1.

The source system (initial microstructure) is conditioned to random bars ordered from south to north in the sense of the dominant direction of the mass diffusion. The target system (reference or final microstructure) is conditioned to a dispersed system randomly distributed that is a homogeneous, periodic, and isotropic CL. Our initial and reference microstructures consider only one filter: the one-point correlation function (pore fraction). The numerical solution considers the black color as the pore phase and the white color as the agglomerate phase. Agglomerates are made up of Pt/C + Nafion.

It must be pointed out that the unknown dimension of the three-dimensional catalyst layer is considered the same as the aligned dimension in our bidimensional simulated microstructure. In this work the aligned dimension is the vertical south-north direction. On this geometrical point of view, the 3D image is extruded along the unknown direction of our 2D images. On this geometrical assumption, and with the knowledge that the oxygen diffusion coefficient is a directional and intensive property, we can define that our two-dimensional values of the effective diffusion coefficient are the same than the extruded three-dimensional structure. There are three sections in Fig. 1: (1) Top section is for user-defined input that includes the initial and final microstructures of the SA method, CL microstructures are fixed to $10\% < \emptyset < 50\%$ (with steps of 10%). Surface fractions higher than 50% were not analyzed because experimentally a higher porosity implies a weak microstructure. (2) Left section describes the SA reconstruction strategy for generating the proposed universe of microstructure meshes. (3) Right section shows the numerical outputs that are reported below.

### Simulated annealing

The SA methodology is an optimization method used to determine local or global optima in complex problems^39,40^. In the iteration of the SA method, a pixel of a different phase is exchanged, and the exchange is evaluated and accepted based on a cooling probability based on a fictitious temperature. This approach transforms the original system into a target system. This technique has been extensively employed in reconstructing stochastic heterogeneous materials (SHM)^32–34,41–43^, demonstrating its versatility and effectiveness. However, it is essential to define some fundamental bases.

The statistical analysis considers the generation of an ensemble (Ω) composed of W realizations *ꞷ;* in this work, W = 15. Statistically, the realization ꞷ represents the domain ${\mathcal{V}}$ of the studied system (CL) within the measurable space $\Re^{d}$, partitioned by a *j* phase. In this context, the region in the space ${\mathcal{V}}_{j} \left( \omega \right),{ }$ the surface fraction $\phi_{j}$($\omega )$, and the index function $I_{j} \left( \omega \right)$ can be defined.

1 $$I_{j} \left( \omega \right) = \left\{ {\begin{array}{*{20}l} {1,} \hfill & { if\; the\; position \left( {x,y} \right) \in {\mathcal{V}}_{j} \left( \omega \right).} \hfill \\ {0,} \hfill & {otherwise,} \hfill \\ \end{array} } \right.$$

The realization ω can be interpreted as a CL of the set Ω of CL´s. All these are microstructures with a finite number of nodes on the x-axis ($Nx$) and the y-axis ($Ny$); the specific phase of each node can be identified. This work studies two-phase isometric materials in a square domain of $Nx = Ny$ pixels. In this way, it is possible to represent the agglomerate matrix phase (white pixels) and the pore dispersion phase (black pixels). When examining the white phase, the index function 1 can be rewritten as:

2 $$I_{j} \left( \omega \right) = \left\{ {\begin{array}{*{20}l} {1,} \hfill & { if\; the\; position\; pixel \left( {x,y} \right) is \;white \left( {agglomerate \;matrix} \right). } \hfill \\ {0,} \hfill & {otherwise \;it\; is\; black \left( {pore\; dispertion} \right). } \hfill \\ \end{array} } \right.$$

Once the domain has been defined, applying different correlation functions to obtain statistical information to characterize the material is possible. In this work, the two-point correlation functions $(S_{j}^{\left( 2 \right)} \left( r \right)$) and the linear path correlation function ($P_{j}$) were employed in both phases of SA reconstruction.

Considering a material with domain ${\mathcal{V}} \subseteq \Re^{d}$, volume V, and composed of *j* = *2* disjoint random phases. The $S_{j}^{\left( 2 \right)} \left( r \right)$ function is the probability that the initial point and the endpoint of a line of length *r* fall in the same phase *j*. Considering an isotropic and homogeneous medium. The $S_{j}^{\left( 2 \right)} \left( r \right)$ can be defined as a function of distance r^28^:

3 $$S_{j}^{\left( 2 \right)} \left( r \right) = {\text{I}}_{j} \left( x \right) I_{j} \left( {x + r} \right),$$

where 〈〉 refers to the statistical average from evaluating the whole domain ꞷ. ${\text{I}}_{J}$ is the index function of the computational domain (Eqs. 1 or 2). The function $S_{j}^{\left( 2 \right)} \left( r \right)$ provides a measure of how the endpoints of a vector r in the studied phase are correlated. On the other hand, the $P_{j}$ function is defined by:

4 $$P_{j} = { }\smallint I_{j} \left( { x + ar} \right)da$$

Both correlation functions are used for the SA reconstruction; they are considered for both phases ($$j_{0}$$ and $$j_{1}$$). Equation (5) defines the error between the source system and the current system ($$E_{SA} )$$:

5 $$E_{SA} = { }\mathop \sum \limits_{r} \left( {F^{\prime } \left( r \right) - F\left( r \right)} \right)^{2}$$

where,

6 $$F\left( r \right) = \frac{1}{4}(\sum S_{j1} \left( r \right) + \sum S_{j2} \left( r \right) + \sum P_{j1} \left( r \right) + \sum P_{j2} \left( r \right))$$

$F\left( r \right)$ characterizes the source system, and $F^{\prime}\left( r \right)$ characterizes the SA current system. The pixel phase shift is accepted if the probability $P\left( {\Delta E_{SA} } \right)$ is satisfied,

7 $$P\left( {\Delta E_{SA} } \right) = \left( {\begin{array}{*{20}l} {1,} \hfill & {\Delta E_{SA} \le 0,} \hfill \\ {\exp \left( {\frac{{ - \Delta E_{SA} }}{T}} \right),} \hfill & {\Delta E_{SA} > 0} \hfill \\ \end{array} } \right)$$

In this work, the SA methodology is applied pixel by pixel, simplifying the theoretical study of the evolution of an initially bar-ordered system as its transitions to a random dispersion system. It should be noted that the user defines both the initial system (ordered bars) and the final system (random dispersion).

### Isotropy

The isotropy of a material can be expressed as a function of the orientation of the tensor describing the transport phenomenon^44^ or as a geometric constraint influencing the covariance matrix in a random field^45^. The relationship between the sum of covariance models in factor spaces and zonal isotropy and the relationship between geometric isotropy and an isotropic covariance function are relevant aspects in this context^46^. It is important to note that geometric and zonal isotropy are interrelated.

The concept of geometric isotropy is a valuable tool for describing the behavior of microstructures based on both accurate models, such as images acquired through scanning electron microscopy and synthetic CLs. This approach for describing microstructures is applicable to CL structures of varying dimensions. However, one of the fundamental challenges is the selection of appropriate scaling factors, which is a significant hurdle to overcome in the field of research. In this study, we will use the $S_{j}^{\left( 2 \right)} \left( r \right)$ to generate a vertical axis representation $S_{j,y}^{\left( 2 \right)} \left( r \right)$ versus the horizontal deviation $S_{j,x}^{\left( 2 \right)} \left( r \right),$

8 $$E_{xy} = { }\frac{1}{n}\sum \left( {S_{j,y} \left( r \right) - S_{j,x} \left( r \right)} \right)^{2}$$

where, $E_{xy}$ is the mean square error of the correlation functions. Since the user defines the initial and final microstructure of the study, it is feasible to normalize the difference for a generalized comparison. The proposed isotropy $(I_{xy}$) is shown in Eq. (9).

9 $$I_{xy} = 1 - \frac{{E_{xy,actual} }}{{E_{xy, initial} { }}}$$

$E_{xy, initial}$ corresponds to the source system of ordered bars (higher anisotropy) and $E_{xy,actual}$ is the mean of the difference between squares $E_{xy}$ of the actual SA iteration system. The bar microstructure is represented by $I_{xy} = 0$, as SA shakes the system $I_{xy}$ tends to 1^38^.

### Effective diffusion coefficient

The solution of the FVM is iterative and requires the implementation of the physical model in the nodal point^46,47^. The molar flux due to diffusion can be calculated from the concentration gradient and a diffusivity coefficient, as are indicated by the first Fick's law described below^48^:

10 $${\mathcal{J}}_{F} = - D \frac{\Delta C}{{\Delta x}}$$

where ${\mathcal{J}}_{F}$ is the flux density of the species diffusing through a unit area, $D$ is the diffusion coefficient, $\Delta C$ is the differential of the species concentration, and $\Delta x$ is the diffusion length. Considering the general equation of transport phenomena described in classical fluid mechanics and that the study is limited to a stationary diffusion state, without a convective and transient phenomenon, as well as without generation or consumption of species, the diffusion in a volume can be expressed as in Eq. (11):

11 $$\mathop \smallint \limits_{SC}^{ } {\text{n}} \cdot \left( {D\nabla C} \right) dA$$

Using the Gaussian divergence theorem, we have that the integration over the CV is equal to the integration over the surface bounding that CV. For two-dimension mesh, the diffusion can be expressed as follows:

12 $$\left( {\frac{{D_{e} }}{{\partial x_{{{\mathcal{P}}E}} }}A_{e} + \frac{{D_{w} }}{{\partial x_{{W{\mathcal{P}}}} }}A_{w} + \frac{{D_{s} }}{{\partial y_{{{\mathcal{P}}S}} }}A_{s} + \frac{{D_{n} }}{{\partial x_{{{\mathcal{P}}N}} }}A_{n} } \right)C_{{\mathcal{P}}} - \left( {\frac{{D_{e} }}{{\partial x_{{{\mathcal{P}}E}} }}A_{e} } \right)C_{E} - \left( {\frac{{D_{w} }}{{\partial x_{{W{\mathcal{P}}}} }}A_{w} } \right)C_{W} - \left( {\frac{{D_{s} }}{{\partial x_{{{\mathcal{P}}S}} }}A_{s} } \right)C_{S} - \left( {\frac{{D_{n} }}{{\partial x_{{{\mathcal{P}}N}} }}A_{n} } \right)C_{N} = 0$$

where $A$ is the CV cross-sectional area, $\partial x$ is the distance of the volume or control element between boundaries, $C$ is the species concentration, $D$ the phase diffusivity coefficient as a function of the flow direction, the subindices $e$, $w$,* s*, and $n$ are the east, west, south, and north direction, respectively. As well as ${\mathcal{P}}$ is the VC central position. The tri-diagonal matrix algorithm (TDMA) is used to determine the pattern of the complete spatial concentration distribution ($C_{x,y}$).

In this study, we define the effective flux (${\mathcal{J}}_{eff}$) as the sum of the local fluxes across horizontal mesh layers. We then utilize the numerical ${\mathcal{J}}_{eff}$ value and modify the proportionality coefficient in the fundamental Eq. (10), but renaming it to $D_{eff}$, as it is shown in Eq. (13),

13 $$D_{eff} = {\mathcal{J}}_{eff} \frac{Ny}{{\Delta C_{Boundary\; condition} }}$$

**Figure 2:**
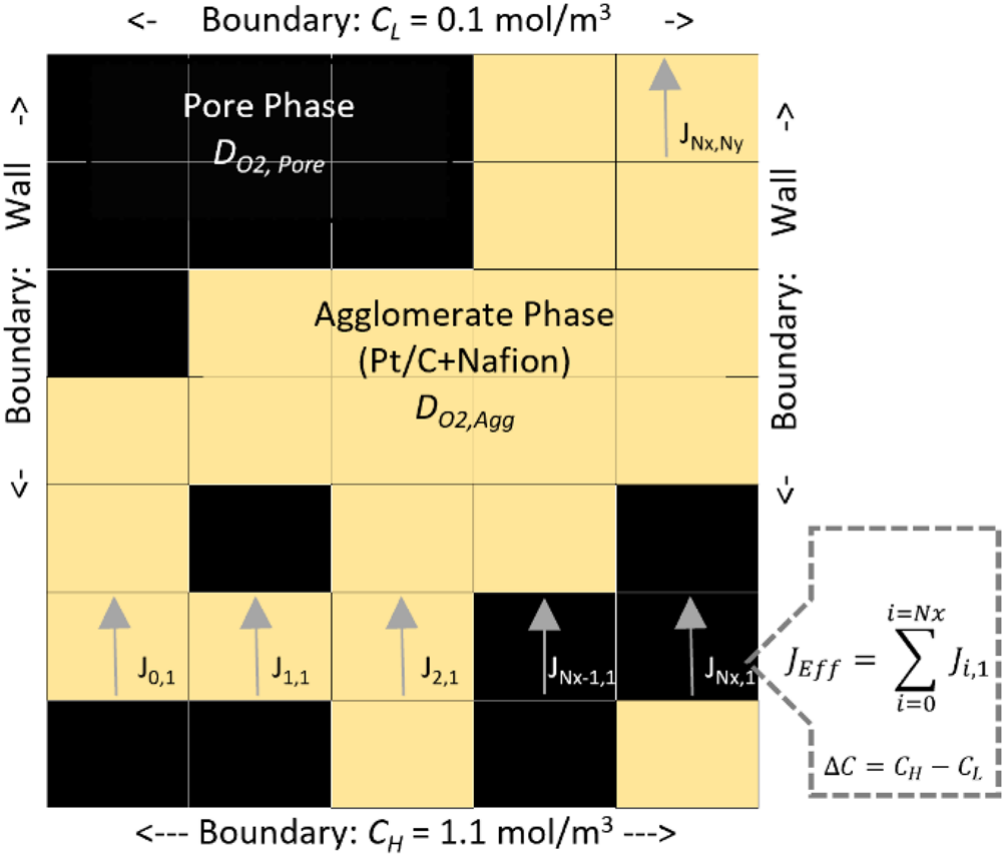
FVM boundary conditions and graphical index of the local fluxes used for the global ${\mathcal{J}}_{Eff}$.

The material properties of the CL mesh define, in a deterministic way, the ETC ($D_{eff}$). Figure 2 shows FVM boundary conditions for ${\mathcal{J}}_{eff}$ numerical determination.

In this work, we simulate both pore (empty medium) and agglomerate phases (Pt/C + Nafion). The diffusion coefficient of oxygen in an empty medium is $D_{O2,Pore} = 3.23{ } \times { }10^{ - 5} { }\;{\text{m}}^{2} \;{\text{s}}^{ - 1}$ and the diffusion coefficient of oxygen in the agglomerate is the same that in the ionomer $D_{O2, Agg} = 8.45{ } \times 10^{ - 9} \;{\text{m}}^{2} \;{\text{s}}^{ - 1}$^49^. The exact analytical solution for a parallel diffusion resistance is given by the Eq. (14).

14 $$D_{0} = \emptyset D_{O2,Pore} + \left( {1 - \emptyset } \right) D_{O2, Agg}$$

where $D_{0}$ is the exact value of the diffusion coefficient when the both phases are vertically aligned ($I_{xy} = 0.0$).

## Results

The process of reconstruction of the catalytic layers starts with the generation of the synthetic microstructures comprised by the pore phase (black color phase) and the agglomerate (white color phase), the microstructures are bars aligned vertically from north to south and have the following microstructural characteristics: surface fraction ($\phi$) of 50% pore—50% agglomerate, 40% pore—60% agglomerate, 30% pore—70% agglomerate, 20% pore—80% agglomerate and 10% pore—90% agglomerate. The domain of all samples is 350 × 350 pixels where each pixel is equivalent to 0.1 µm, so the samples are 35 µm × 35 µm.

**Figure 3:**
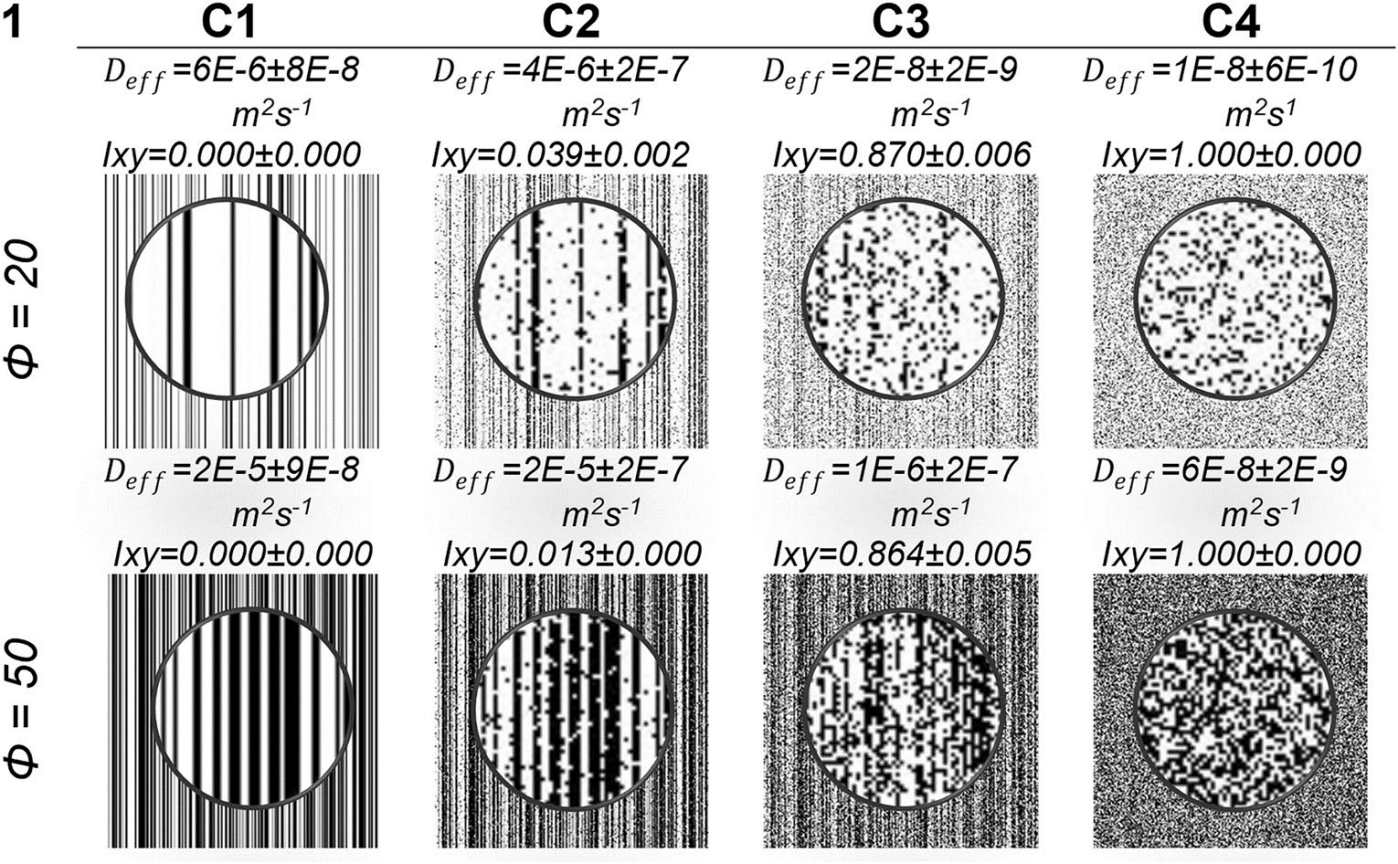
Representative images of the SA process of the studied CL microstructures.

The SA method is employed to obtain an ensemble ${\Omega }$ composed of W = 15 random repetitions for each studied CL. The results present the CL isotropy and the $D_{eff}$ determined for both pore and agglomerate phases. Figure 3 shows representative images of the studied CL microstructures. The first column shows porosity, and the C1 column shows the aligned bars when $I_{SA} = 0$ iteration, C2 column is for $I_{SA} = 5000$ iteration, C3 column is for $I_{SA} = 20000$ iteration, and the C4 column shows the dispersed random material (reconstructed image).

It is important to highlight that all samples' SA reconstruction temperature was the same ( $$T = 1 x 10^{ - 6} )$$ and the reconstructions were stirring until the convergence of the annealing error $$E_{SA} = 1 x 10^{ - 6}$$. The C1 column indicates the bar-ordered CL system, $$I_{xy} = 0.00 \pm 0.00$$, while the C4 column represents the homogeneously agitated CL system $$I_{xy} = 1.00 \pm 0.00$$. C2 and C3 columns were selected to analyze their behavior in the results shown in Fig. 6. Each image in Fig. 3 represents a Ω ensemble, but the values are the averages and standard deviation obtained for W = 15.

**Figure 4:**
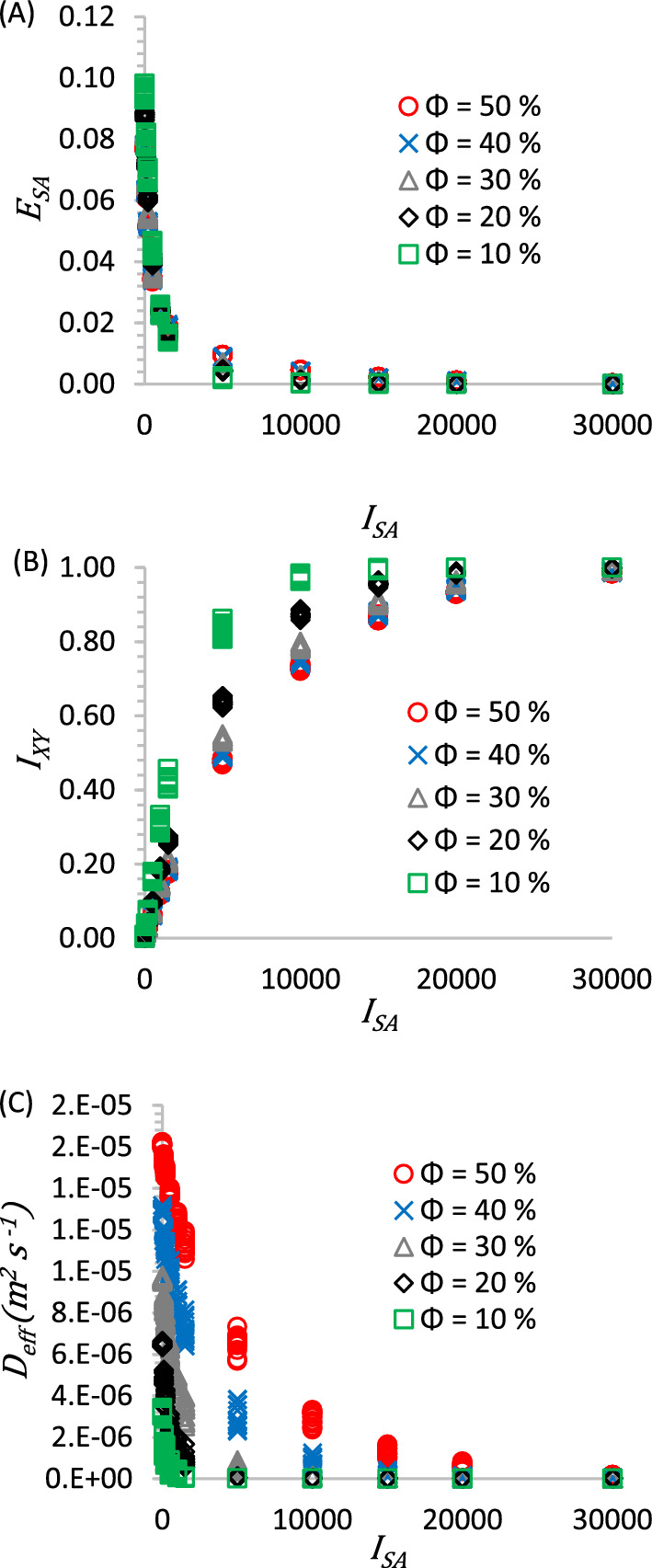
$E_{SA}$ error response (**A**), $I_{xy}$ isotropy response (**B**), and $D_{eff}$ coefficient (**C**), the three graphs as a function of $I_{SA}$ iteration.

Figure 4 presents plots based on $I_{SA}$ iterations: (A) for the error variation of the reconstruction process $E_{SA}$ (Eq. 5), (B) for the isotropy variation $I_{xy}$ (Eq. 9) and (C) for $D_{eff}$ (Eq. 13), all graphs based on the $I_{SA}$ statistical iteration. The values presented in this figure include all the universe of ω realizations of the total samples studied.

Figure 4A shows that the $E_{SA}$ value tends to zero, which indicates that the actual microstructure of the CL is converging to the values of the objective microstructure of the CL, as observed in the representative images of Fig. 3. As the $I_{SA}$ iterations increase, the overall error between the reconstructed microstructure and the target microstructure decreases, the reconstruction can be seen in Fig. 3, where in column 1 we have the initial microstructure arranged in aligned bars and in the following columns 2 to 4 the dispersion of the pore phase and the agglomerated phase is observed.

The $I_{xy}$ values in Fig. 4B tend to unity, indicating that the error between the correlation functions $S_{j,x} \left( r \right)$ and $S_{j,y} \left( r \right)$ are decreasing, as indicated in Eq. (9). It is observed that $I_{xy}$ is more dependent of Φ than $E_{SA}$, this trend is similar to that reported by^38^. Figure 4C shows the response of $D_{eff}$ coefficient depending on the reconstruction process iteration $I_{SA}$. It is observed that the $D_{eff}$ variation is higher for the first $I_{SA}$ iterations.

**Figure 5:**
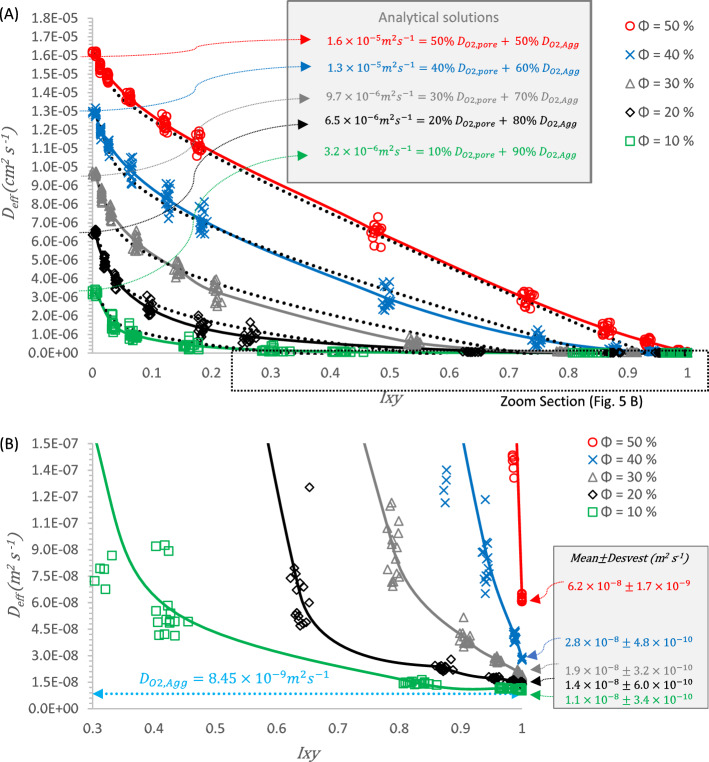
$D_{eff}$ coefficient as a function of the isotropy evolution $I_{xy}$: (**A**) for all possibilities and analytical solutions magnitudes @ $I_{xy} = 0$ and (**B**) for a zoom section and numerical results @ $I_{xy} = 1$.

Figure 5 shows the response of $D_{eff}$ compared to the evolution of isotropy $I_{xy}$. It must be pointed out that the numerical solution considers the black color as the pore phase and the white color as the agglomerate phase, both for a synthesized microstructure of a catalytic layer. The values presented in this figure includes all possibilities (points), average values (solid lines) and the curves of $D_{eff}$ after solving the new proposed Eq. (15) (black dotted lines).

Figure 5A shows the evolution of $D_{eff}$ as a function of isotropy $I_{xy}$. The results from all examined samples (${\Omega } \in {\text{W}} = 15{ }\omega$) are presented with markers and solid lines denoting their averages. It can be observed that when the $I_{xy} = 0$ the microstructure corresponds to the pore phase and the agglomerate are vertically aligned. In this condition, it is observed that $D_{eff}$ values are equal to the analytical solution of a parallel diffusion resistances; these values their equations are highlighted in the gray shading of Fig. 5A. The values of $D_{0}$ obtained by exact analytical solution of Eq. (14) are: $D_{0} \left( {\emptyset = 50} \right) = 1.6 \times 10^{ - 5} \;{\text{m}}^{2} \;{\text{s}}^{ - 1}$@ (50%$D_{O2,Pore}$—50%$D_{O2,Agg}$) and $D_{0} \left( {\emptyset = 10} \right) = 3.2 \times 10^{ - 6} \;{\text{m}}^{2} \;{\text{s}}^{ - 1}$@ (10%$D_{O2,Pore}$—90%$D_{O2,Agg}$). The theoretically based Eq. (15) is proposed for the range $D_{eff} \ge 5\% D_{0}$. This range is selected by the authors to reduce the variance between the numerical average values and the analytical solution values.

15 $$\frac{{D_{eff} }}{{D_{0} }} = \left\{ {\begin{array}{*{20}l} {\frac{{D_{eff} }}{{D_{0} }} = 1 - m_{1} I_{xy} - m_{2} ln\left( {\frac{{I_{xy} }}{{I_{{xy_{0} }} }}} \right),} \hfill & {if\; D_{eff} \ge 5\% D_{0} } \hfill \\ {Numerical \;solution, } \hfill & {if \;D_{eff} < 5\% D_{0} } \hfill \\ \end{array} } \right.$$

where $D_{0}$ is the exact solution of Eq. (14), $I_{{xy_{0} }}$ is the lowest numerical $I_{xy}$ value different to cero (in this work $I_{{xy_{0} }} = 5.40 \times 10^{ - 3}$) and $m_{1}$ y $m_{2}$ are analytical slopes obtained from numerical data, related by the porosity $\emptyset$ in Eqs. (16) and (17).

16 $$m_{1} = 1.9 \emptyset - 0.19$$

17 $$m_{2} = - 0.48 \emptyset + 0.29$$

Table 1 shows the solution of Eqs. (14, 16 and 17). These values are applied in Eq. (15) to obtain the black dotted lines in Fig. 5A.

**Table 1:** Numerical values used in equation 15.

Porosity ∅ (%)	$D_{0}$(m^2^ s^−1^)	*m*_1_	*m*_2_
10	3.24E−06	0.00	0.24
20	6.47E−06	0.19	0.19
30	9.70E−06	0.38	0.15
40	1.29E−05	0.57	0.10
50	1.62E−05	0.76	0.05

Ceballos et al.^30^ reports effective oxygen diffusion values for a catalytic layer of a PEMFC with 30% pore phase surface fraction; the results reported in their work are obtained by solving different effective diffusion models, they report oxygen $D_{eff}$ in a range of $1.87 \times 10^{ - 6} \; {\text{m}}^{2} \;{\text{s}}^{ - 1}$ for Nam and Kabiany model and $8.91 \times 10^{ - 6} \;{\text{m}}^{2} \;{\text{s}}^{ - 1}$ for Tomadakis and Storirchos model; In this work, for the same pore phase surface fraction condition an analytical result of $9.7 \times 10^{ - 6} \;{\text{m}}^{2} \;{\text{s}}^{ - 1}$ is obtained. The variations in the reported values are attributed to the different microstructures of the evaluated electrodes.

As isotropy increases, $D_{eff}$ values exhibit an exponential decrease, the exponential decrease being particularly pronounced in the sample $\phi = 10 \%$ (green color) and less pronounced in the sample $\phi = 50 \%$ (red color). Figure 5B shows a zoom section for details of the range $D_{eff} \le 1.5 \times 10^{ - 7} \; {\text{m}}^{2} \;{\text{s}}^{ - 1}$ and $I_{xy} \ge 0.3$. In this figure, the diffusion coefficient of oxygen in the agglomerate (data input: $D_{O2, Agg} = 8.45 \times { }10^{ - 9} \; {\text{m}}^{2} \;{\text{s}}^{ - 1}$) is noted by the dashed blue line.

Zhao et al.^50^ report experimentally obtained magnitudes of the effective diffusivity coefficient for catalytic layers. The reported averages of $D_{eff}$ for catalytic layers range from $4.2 \pm 0.9 \times 10^{ - 7} \; {\text{m}}^{2} \;{\text{s}}^{ - 1}$ @ 25 °C to $4.6 \pm 0.5 \times 10^{ - 7} \;{\text{m}}^{2} \;{\text{s}}^{ - 1}$ @ 75 °C. Experimental samples exhibited a surface fraction of the pore phase within a range of $\phi$ = 30% to $\phi$ = 40%. In this study, the reported values for different surface fractions of the pore phase and agglomerates are in the range of magnitudes of the order of $10^{ - 6}$ when $I_{xy}$ is equal to zero and $10^{ - 8}$ when $I_{xy}$ is equal to one. Our numerical model takes into account the diffusive coefficient of oxygen within the agglomerate, attributing that as the pore phase decreases the $D_{eff}$ values approach the reported value of $D_{O2, Agg} = 8.45{ } \times { }10^{ - 9} \;{\text{m}}^{2} \;{\text{s}}^{ - 1} { }$. Similarly, Shen et al.^51^ report experimentally obtained magnitudes of $D_{eff}$ for a catalytic layer with different thicknesses; the average value obtained is $1.47 \pm 0.05 \times 10^{ - 7} \;{\text{m}}^{2} \;{\text{s}}^{ - 1}$, a figure that also falls within the range of results obtained in this study. Chen et al.^52^ presents results of the effective diffusivity coefficient of oxygen for a sample with a surface fraction of 17% for the pore phase using the lattice Boltzmann method, reporting a $D_{eff}$ of $7.71 \times 10^{ - 8} \;{\text{m}}^{2} \;{\text{s}}^{ - 1}$. In our work, the result obtained for a similar surface fraction of the pore phase is $1.4 \times 10^{ - 8} \;{\text{m}}^{2} \;{\text{s}}^{ - 1}$ when it is 20% $D_{O2,Pore}$- 80% $D_{O2,Agg}$. The variation in results is attributed to the difference in the isotropy value between synthetic and experimental materials. Microstructural isotropy directly influences the behavior of the effective diffusivity coefficient. The results show that the exponential tendency is preserved at higher isotropy ranges. The box highlighted in the gray shading of Fig. 5B presents $D_{eff} @ I_{xy} = 1$.

As evidenced by the results, the impact of oxygen diffusion within the agglomerate exhibits insignificance concerning the effective diffusion coefficient, $D_{eff}$, particularly when the surface fraction of the porous phase is predominant. Conversely, in scenarios where the surface fraction of the agglomerate phase predominates, it becomes apparent that the diffusion within the agglomerate significantly influences the ultimate determination of the diffusion coefficient. This observation is elucidated in the zoom section in Fig. 5.

**Figure 6:**
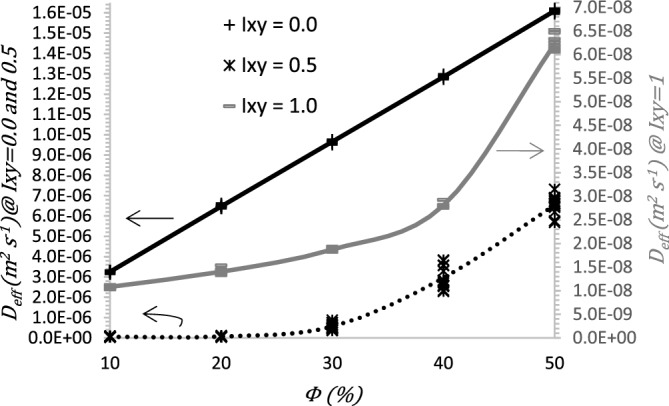
$D_{eff}$ coefficient as a function of agglomerate surface fraction $\phi$, for different values of isotropy $I_{xy}$.

Figure 6 shows $D_{eff}$ as a function of pore surface fraction $\phi$, for three selected isotropies referred to in columns of Figs. 3 and 5: the solid black line for $I_{xy} = 0.0$, the gay line for $I_{xy} = 1.0{ }$ and dashed black line for $I_{xy} = 0.5$.

Figure 6 shows the results obtained for $D_{eff}$ as a function of the superficial fraction of the agglomerate phase. The results from all examined samples (${\Omega } \in {\text{W}} = 15{ }\omega$) are presented with markers and solid lines denoting their averages. For $I_{xy}$ = 0.0 and $I_{xy}$ = 0.5 the $D_{eff}$ ranges are on the left side of the graph; for $I_{xy}$ = 1.0 the $D_{eff}$ ranges are on the right side of the graph.

The results are presented within $\phi$ = 10% to $\phi$ = 50%, with 10% intervals. The solid black line, labeled as $I_{xy}$ = 0.0, corresponds to the average of all samples when the microstructure is vertically aligned. Numerical results indicate that the effective diffusion coefficient is directly related to the porosity of the catalyst layer microstructure. Higher porosity has a positive impact on $D_{eff}$ enhancement, this trend agrees with empirical correlation models of the effective diffusion coefficient in porous materials (Bruggeman^53^, Neale and Nader^54^, Tomadakis and Sotirchos^55^, Mezedur et al.^56^, Zamel et al.^57^, Das et al.^58^). The results suggest that the isotropy of the microstructure also have a significant effect on the $D_{eff}$ as shown in Fig. 6. An increasing trend is observed when the porosity of the microstructure is more significant. When the porosity is minor, the diffusion of the agglomerate becomes more relevant in the effective diffusion coefficient, because the higher the surface fraction of the agglomerate, the higher the value of the predominant oxygen diffusion is that of the diffusion in the ionomer, which can be seen in Fig. 6^50,59^ $D_{eff}$ values range from 3.26 × 10^–6^ m^2^ s^−1^@ $\phi$ = 10% to 1.60 × 10^–5^ m^2^ s^−1^@ $\phi$ = 50%. This behavior is attributed to the reduced diffusive resistance when the phases are vertically aligned. As isotropy in the microstructure increases, $D_{eff}$ diminishes due to the agitation within the microstructure. When $I_{xy}$ = 0.5, $D_{eff}$ values are within the range of 3.74 × 10^–8^ m^2^ s^−1^@ $\phi$ = 10% and 6.53 × 10^–6^ m^2^ s^−1^@ $\phi$ = 50%. In the case of a randomly monodisperse microstructure ($I_{xy}$ = 1.0), the value of $D_{eff}$ varies between 1.08 × 10^–8^ m^2^ s^−1^@ $\phi$ = 10% and 6.22 × 10^–8^ m^2^ s^−1^@ $\phi$ = 50%. Accordingly, microstructures with lower isotropy (aligned bars) and higher porosity have lower diffusion resistance, which translates as higher $D_{eff}$.

## Conclusions

In this study, a theorical investigation has been carried out to determine the effect of the isotropy on the effective diffusion coefficient of PEMFC catalyst layers that change from aligned to dispersed pattern microstructure. The CL´s studied in this work are composed of two diffusive phases, the porosity, and agglomerated phases. We apply two advanced numerical methodologies: (1) Finite volume method to define a specific oxygen diffusion coefficient into each mesh node: $D_{O2,Pore}$ for pores and $D_{O2,Agg}$ for agglomerates. (2) Simulated Annealing to simulate the evolution of unidirectional aligned bars to random mono-dispersed CL´s.

The analysis confirms that the aligned bar is the better structure for improving the CL mass transport phenomena when the diffusion is made in the parallel direction to the bars because there is less resistance to the transport of the oxygen molecule. Further, this statistical strategy allows us to determine the effect of geometric isotropy ($I_{xy}$) on the effective diffusion coefficient ($D_{eff}$). When the superficial fraction of the pore and the agglomerate are both equal to 50% in a monodisperse random configuration ($I_{xy}$ = 1.0 + 0.0), $D_{eff}$ exhibits values within a range with a maximum of 6.20 × 10^–8^ m^2^ s^−1^ and a minimum close to the oxygen diffusion coefficient in the ionomer. As the superficial fraction of the pore decreases, the value of $D_{eff}$ decreases significantly. For instance, when the superficial fraction of the pore phase is 10% values close to the diffusion coefficient of oxygen in the ionomer are obtained. As the surface fraction of the agglomerate is predominant, the $D_{eff}$ is mostly influenced by the $D_{O2,Agg}$, which impacts the overall performance of the fuel cell.

In conclusion, it has been observed that in samples with a surface fraction of the porous phase equal or higher than 50% at isotropy = 0, anisotropic material, the effective diffusion coefficient presents a higher value than when the isotropy is equal to 1. This decrease is attributed to the dispersion of the phases by the SA agitation process, the diffusion coefficient decreases exponentially until reaching a maximum of isotropy. At surface fractions greater than or equal to 50% of the pore phase the diffusion coefficient is influenced by the diffusion of oxygen in the pore, since it is the predominant phase.

On the other hand, as isotropic values approach unity, a change in the behavior of the effective diffusion is observed, with oxygen diffusion in the agglomerate being the predominant factor. This change becomes more evident in samples with porous phase surface fractions below 50%, where the effective diffusion coefficient shows an exponential decrease as the isotropy process progresses.

Ultimately, as the isotropic values approach unity, the effective diffusion coefficient falls into ranges close to the diffusion of oxygen in the ionomer, indicating that this phase becomes predominant in the microstructure. These findings are relevant to understanding and controlling diffusion processes in anisotropic materials, providing valuable information for the design and optimization of devices and systems that rely on the diffusion species.

The results obtained in this study provide information on the behavior of the Diffusion coefficients in different porosity conditions and how this directly affects the effective transport coefficient. In turn, it provides a methodology that is aimed at a better design and manufacture of catalytic layers of PEM fuel cells, leading to a better overall performance of the cell.
